# Biophysical characterization and modeling of human Ecdysoneless (ECD) protein supports a scaffolding function

**DOI:** 10.3934/biophy.2016.1.195

**Published:** 2016-03-09

**Authors:** Riyaz A. Mir, Jeff Lovelace, Nicholas P. Schafer, Peter D. Simone, Admir Kellezi, Carol Kolar, Gaelle Spagnol, Paul L. Sorgen, Hamid Band, Vimla Band, Gloria E. O. Borgstahl

**Affiliations:** 1 Department of Genetics, Cell Biology and Anatomy, University of Nebraska Medical Center, Omaha, NE 68198, USA; 2 Eppley Institute for Research in Cancer and Allied Diseases, University of Nebraska Medical Center, Omaha, NE 68198, USA; 3 Interdisciplinary Nanoscience Center (iNANO), Aarhus University, Gustav Wieds Vej 14, 8000 Aarhus C, Denmark; 4 Department of Biochemistry & Molecular Biology, University of Nebraska Medical Center, Omaha, NE 68198, USA

**Keywords:** Ecdysoneless, circular dichroism, SAXS, molecular modeling, structural hub, scaffold protein

## Abstract

The human homolog of Drosophila ecdysoneless protein (ECD) is a p53 binding protein that stabilizes and enhances p53 functions. Homozygous deletion of mouse *Ecd* is early embryonic lethal and *Ecd* deletion delays G_1_-S cell cycle progression. Importantly, ECD directly interacts with the Rb tumor suppressor and competes with the E2F transcription factor for binding to Rb. Further studies demonstrated ECD is overexpressed in breast and pancreatic cancers and its overexpression correlates with poor patient survival. ECD overexpression together with Ras induces cellular transformation through upregulation of autophagy. Recently we demonstrated that CK2 mediated phosphorylation of ECD and interaction with R2TP complex are important for its cell cycle regulatory function. Considering that ECD is a component of multiprotein complexes and its crystal structure is unknown, we characterized ECD structure by circular dichroism measurements and sequence analysis software. These analyses suggest that the majority of ECD is composed of α-helices. Furthermore, small angle X-ray scattering (SAXS) analysis showed that deletion fragments, ECD(1–432) and ECD(1–534), are both well-folded and reveals that the first 400 residues are globular and the next 100 residues are in an extended cylindrical structure. Taking all these results together, we speculate that ECD acts like a structural hub or scaffolding protein in its association with its protein partners. In the future, the hypothetical model presented here for ECD will need to be tested experimentally.

## 1. Introduction

Precisely regulated cell proliferation is essential for embryonic development as well as homeostasis in adult organs and tissues, whereas uncontrolled cell proliferation is a hallmark of cancer [1]. Thus, understanding how the cell cycle machinery is controlled is an important area of research in normal development and cancer cell biology. Extensive research has led to current models of cell cycle control [2]. In quiescent cells, E2F transcription factors are repressed by their association with hypo-phosphorylated Retinoblastoma (Rb) protein family members [3]. A key mechanism to switch E2Fs from a repressed state to an active state is the phosphorylation of Rb protein by cell cycle-associated cyclin-dependent kinases (CDKs) [3]. This results in the disruption of Rb-E2F complexes due to reduced interaction of E2Fs with hyper-phosphorylated Rb. Transcription of E2F target genes is required for cell cycle progression [4].

Our previous studies show the human homolog of Drosophila ecdysoneless protein (ECD) as a novel Rb binding partner [5]. The ecdysone hormone is responsible for coordinating embryogenesis, larval molting and metamorphosis. The “ecdysoneless” phenotype of Drosophila has been known for decades, is caused by “*Ecd*” mutations and emanates from reduced secretion of ecdysone [6]. Only recently however did the cloning of Drosophila *Ecd* gene lead to an appreciation that the ECD protein plays a cell-autonomous role in development and oogenesis separate from its role in ecdysone production [7].

Homozygous *Ecd* deletion in mice is early embryonic lethal and ex vivo Cre-mediated *Ecd* deletion in mouse embryonic fibroblasts (MEFs) or knockdown in human epithelial cells induces cell cycle arrest [5], implicating ECD as a novel cell cycle regulator. We demonstrated that the C-terminus of ECD directly binds to retinoblastoma (Rb) protein at the pocket domain, and competes with E2F for binding to hypo-phosphorylated Rb [5]. ECD binds to p53, stabilizes it and enhances p53 function when overexpressed [8]. Further studies demonstrated that ECD is overexpressed in breast [9], and pancreatic [10] cancers; and its overexpression correlates with poor survival in breast cancer patients [9]. Furthermore, co-overexpression of ECD with mutant Ras rendered immortal epithelial cells fully tumorigenic, through mechanisms that involve an elevation of the autophagy program [11].

Recent studies demonstrate ECD phosphorylation on two serine residues by Casein Kinase-2 (CK2) creates a motif for binding of PIH1D1 (also called NOP17), a component of the HSP90-associated co-chaperone R2TP complex [12]. The R2TP complex is composed of PIH1D1, RPAP3 (also known as hSPAGH), RUVBL1 (has multiple names-Pontin, RVB1, TIP49A, TAP54alpha, ECP-54, TIH1, p50) and RUVBL2 (also known as Reptin, RVB2, TIP49B, TAP54 beta, ECP-51, TIH2, p47) proteins [13], and is involved in the assembly of large protein-protein or protein-RNA complexes, such as RNA polymerase, small nucleolar ribonucleoproteins (snoRNPs), and phosphatidylinositol 3-kinase-related kinases (PIKKs) [14,15]. Recent data shows that the N-terminus of ECD independently associates with RUVBL1, and this interaction is required for its cell cycle regulatory function [16]. Phosphorylation of six ECD serine residues is important for its cell cycle regulation function [16]. ECD has also been shown to interact with TXNIP and helps in p53 stabilization [17]. A recent study by Claudius et al demonstrated that human ECD can rescue splicing defects in drosophila induced by deletion of fly Ecd and Ecd also interacts with a complex containing Prp8, Aar2, Brr2 and Snu114 proteins [18]. The interaction of human ECD with human PRPF8 has been confirmed [16].

Here we have characterized ECD protein structure by circular dichroism measurements and sequence analysis software. These analyses suggest that the majority of ECD is composed of α-helices and that the C-terminal 100 or so amino acids are disordered in the absence of binding partners. Small angle X-ray scattering (SAXS) analysis showed that deletionf ragments, ECD(1–432) and ECD(1–534) are both well-folded and reveals that the first 400 residues are globular and the next 100 residues are in an extended cylindrical structure. The majority of ECD residues are within 30 to 35 Å of each other with a maximal dimension for ECD(1–432) and ECD(1–534) of 92 and 122 Å, respectively. It is noteworthy that the extended C-terminus of ECD contains CK2 sites where PIH1D1 and Rb interact. Finally, a theoretical structural model for ECD (1–432) was calculated that suggests ECD acts like a structural hub or scaffolding protein to associate with its protein partners. This hypothetical model and function of ECD will be tested experimentally in the future.

## 2. Materials and Methods

### 2.1. Protein expression and purification

ECD(1–432) and ECD(1–534) sequences were subcloned into a pET28a vector with a C-terminal 6X-His tag. Plasmids were transformed into BL21(DE3) cells, grown in LB medium at 37 °C to an OD_600_ of 0.7, and induced with 0.5 mM IPTG overnight in an 18°C shaking incubator. Cells were collected by centrifugation, suspended in lysing buffer (2X PBS pH 7.8, 1% Triton X-100, 20 mM imidazole, 2 mM β-mercaptoethanol and protease inhibitor cocktail), lysed using an Emulsiflex-C3 (Avestin, Inc.), and purified by nickel affinity chromatography (5 mL His-Trap FF; GE Healthcare) using an ÄKTAfplc program for a linear gradient from 20 to 400 mM imidazole in basic buffer (2X PBS pH 7.8, 2 mM β-mercaptoethanol). Nearly pure sample eluted at approximately 150 mM imidazole. The product was polished by passing over a Superdex75 HiLoad 16/60 column (GE Healthcare) using isocratic elution into 20 mM HEPES pH 7.5, 250 mM NaCl, 5 mM β-mercaptoethanol. Dynamic light scattering (DynaPro MX/S; Wyatt Technology Corp.) was used to select monodisperse fractions for CD and SAXS experiments. For CD, aliquots of protein were dialyzed into 1X PBS using a 3500 MWCO Slide-A-Lyzer Dialysis Cassette (Thermo Pierce). At all steps, identity and purity of the proteins were assessed by SDS-PAGE analysis.

### 2.2. Circular Dichroism (CD)

CD experiments were performed on a JASCO J-815 CD spectrometer fitted with a Peltier temperature control system (Easton, MD). Each spectrum was the average of five scans collected in the far UV (260–195 nm), with a 0.1 mm path length quartz cell, using a bandwidth of 1 nm, an integration time of 1 sec, and a scan rate of 50 nm/min. All spectra were corrected by subtracting the solvent spectrum acquired under identical conditions. The protein concentration for each sample was between 35 and 45 μM, in 1X PBS. All CD data were processed from mDeg to mean residue ellipiticity (MRE; deg cm^2^ dmol^−1^) using the Spectra Analysis function of Jasco Manager Version 2, to account for the concentration differences. Secondary structure composition of each spectrum was analyzed using the K2D method provided by the online program DichroWeb [19]. Thermal stability data were collected in the far UV at 222 nm (loss of secondary structure), at the same concentrations and in the same condition as for the CD experiments. CD values were recorded every 1 °C from 25 to 80 °C, after the temperature equilibrated for 5 sec at ± 0.1 °C from target temperature. The thermal unfolding temperature was determined by fitting the curve to a Boltzmann sigmoidal equation using a nonlinear least squares fitting algorithm (GraphPad Prism software).

### 2.3. SAXS data collection and processing

Data from monodisperse samples at three concentrations were collected at room temperature on a Rigaku BioSAXS 1000 system with an FR-E rotating anode X-ray generator (λ = 1.54 Å). Concentrations were determined by Bradford protein assays. Images were collected for 90 min and subframes were taken every ten min to examine the data for radiation damage. No radiation damage was observed in the data. The data were processed using the Automated Analysis Pipeline in the SAXSLab software (Rigaku). SAXSLab provides a GUI for the ATSAS Package [20], transparently creating scripts and running the associated ATSAS programs and then presenting the results back to the user. The SAXSLab software automatically subtracts the buffer and calculates the Guinier plot, radius of gyration, molecular weight and volume, pair distribution function, Kratky plot, an infinite dilution scattering curve, and *ab initio* bead model fits to the data. Dmax was calculated from the X-intercept of the P(r) curve. For both the ECD(1–432) and ECD(1–534) proteins, nine bead models were generated from the calculated infinite dilution scattering curves using the slow annealing option. Shannon analysis was performed by Shanum software [21]. Two bead models, one for each protein, with the lowest normalized spatial discrepancy (NSD) value were aligned using the Colores program from the Situs package to dock the smaller ECD(1–432) bead model into the ECD(1–534) bead model [22,23]. This program performs an exhaustive search of the rotational and translational degrees of freedom using the linear cross-correlation as the fitting criterion. Figures were generated with PyMOL (Schrödinger, LLC).

## 3. Results and Discussion

### 3.1. Amino acid sequence analysis, sample preparation and initial characterization

**Figure 1:**
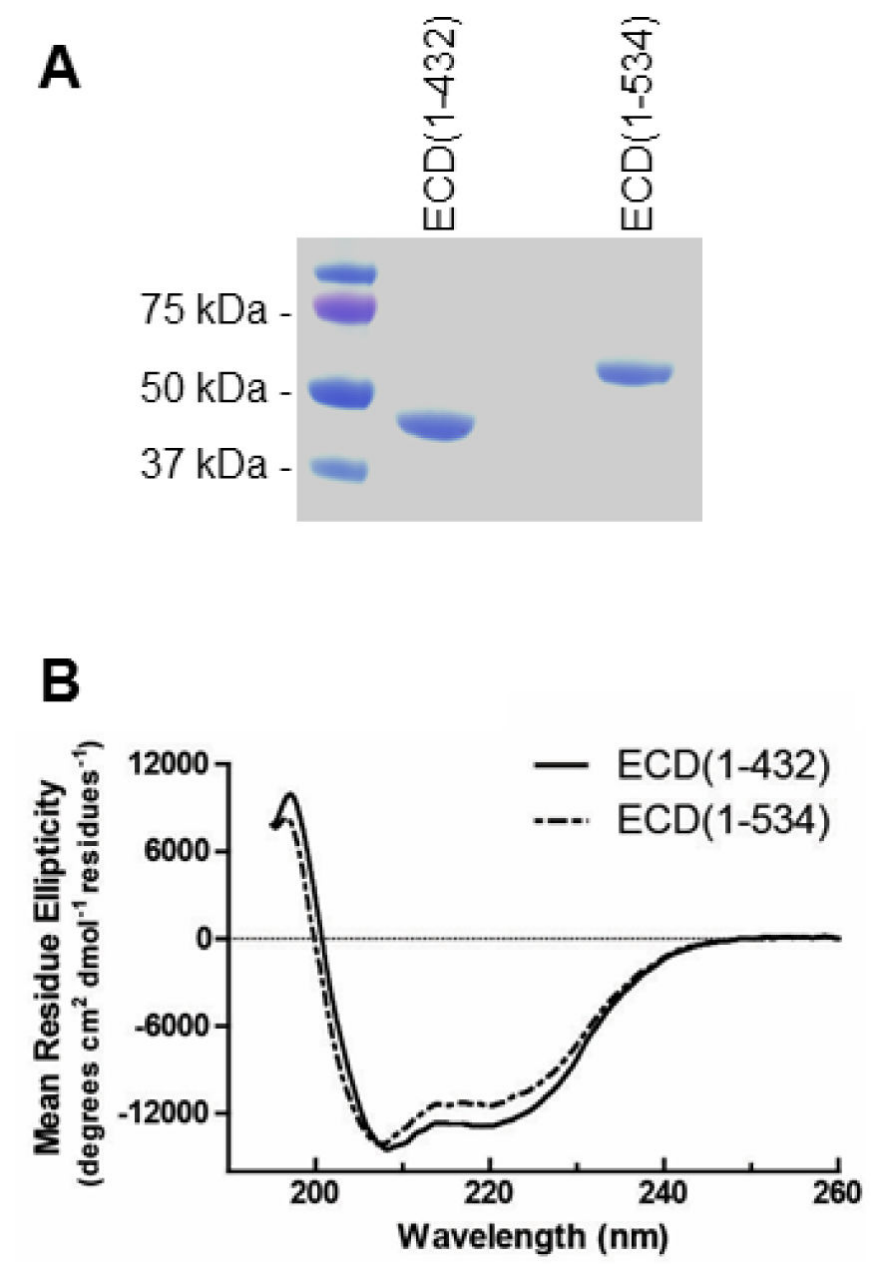
Characterization of ECD(1–432) and ECD(1–534) samples. (A) Purity shown with Coomassie-stained SDS-PAGE. (B) Secondary structure measured with circular dichroism at 25 °C(B).

Given that the three-dimensional structure of ECD is not known, a secondary structure prediction was performed with Protean software from the DNAstar Lasergene 10 core suite (see Supplementary Figure S1 line 1 and line 2). Disordered protein structure was also predicted with PONDR (see Supplementary Figure S1 line 4)[24–26]. Full length ECD(1–644) was predicted to have 60% α-helix. Also, 35% was predicted to be disordered and the most disordered region was in the last 100 C-terminal residues. Based on this information, two deletion fragments, ECD(1–432) and ECD(1–534), were generated. These proteins were purified to homogeneity (Figure 1A) and shown to be monodisperse and of monomeric quaternary structure by dynamic light scattering analysis [27]. Circular dichroism (CD) data were measured (Figure 1B) and fitted with Dichroweb to estimate secondary structure content. ECD(1–432) contained 41% α-helix, 17% β-sheet and 42% random coil, whereas ECD(1–534) contained 38% α-helix, 16% β-sheet and 46% random coil. Predicting protein secondary structure from amino acid sequence calculates that ECD(1–432) is 62.5% α-helix, 3.7% β-sheet, 16.4% turns, and 17.6% coil [28]. However, the CD data indicated less α-helix than the observed and more β-sheet structure, indicating that the Protean prediction software over predicted the amount of α-helix for ECD.

### 3.2. Analysis of protein stability

**Figure 2:**
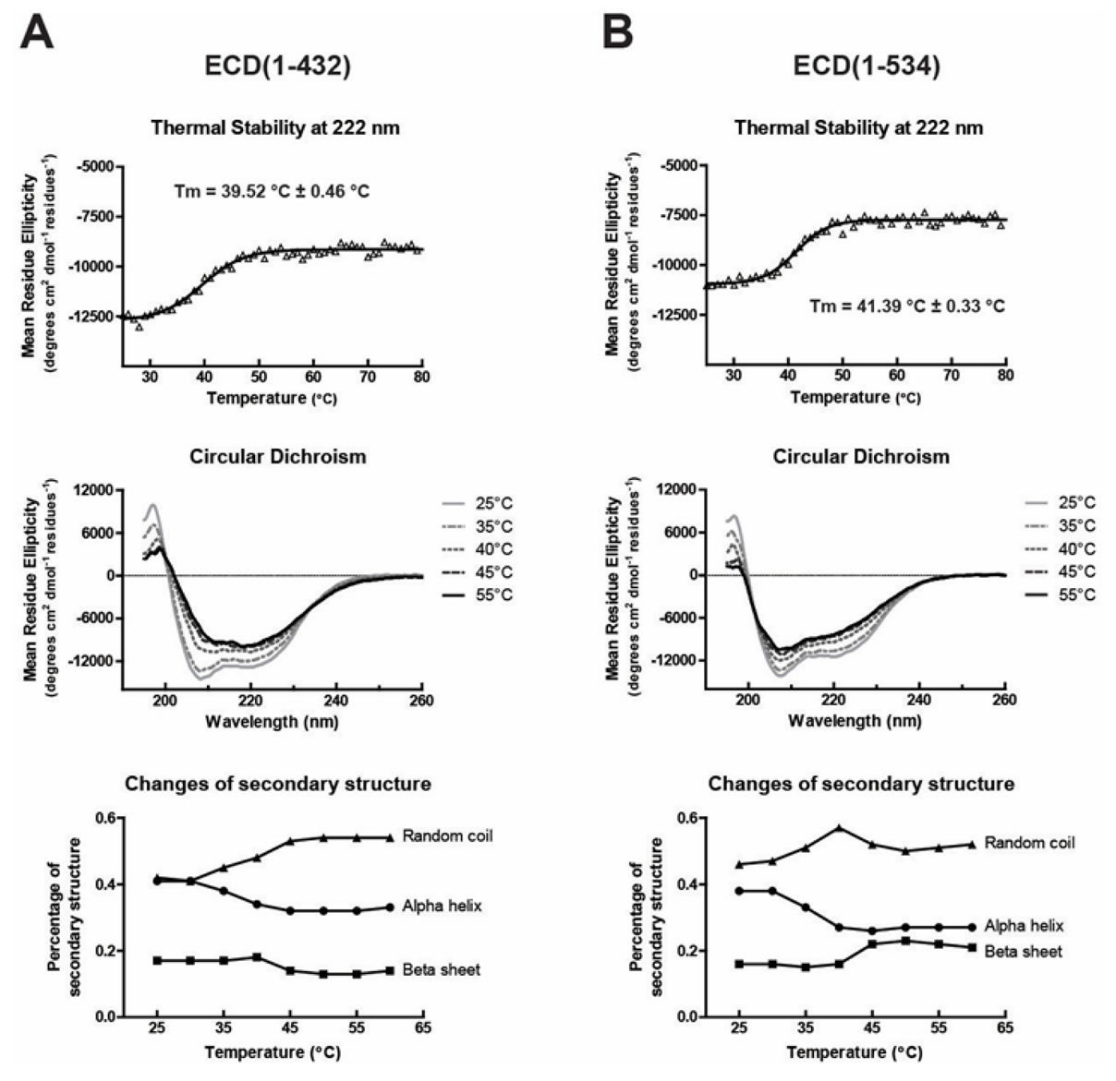
Thermal stability study of ECD fragments by CD. Tm of (A) ECD(1–432) and (B) ECD(1–534) were measured by the change of CD signal at 222 nm from 25 to 80 °C (upper panel). The Tm was determined by nonlinear least squares fitting. In the middle panel, only spectra obtained at 25 (pale grey solid line), 35 (grey dot-dashed line), 40 (grey dotted line), 45 (dark grey dashed line), and 55 °C (black solid line) are displayed for clarity. In the bottom panel, the percentage changes of the random coiled (triangle), α-helix (dot), and β-sheet (square) composition were estimated by DichroWeb and plotted as a function of temperature.

To get an estimate for how tightly ECD is folded we measured the melting temperature (Tm) of ECD(1–432) and ECD(1–534). CD analysis was used to observe the unfolding of ECD. The data indicate that in the absence of binding partners, ECD has a very low Tm with values of 39.5 °C for ECD(1–432) and 41.4 °C for ECD(1–534) (Figure 2). The α-helices and β-strands convert to random coils upon unfolding. These data indicate that the folded domains within the ECD fragments are loosely held together. Limited proteolysis experiment using trypsin on ECD(1–432) showed release of a 10 kDa domain(s) (data not shown). Furthermore, N-terminal amino acid sequencing showed a mixture and indicated that these domains were from both the N-and C-termini of ECD(1–432).

### 3.3. Measurement of 3D structures at low resolution

**Figure 3:**
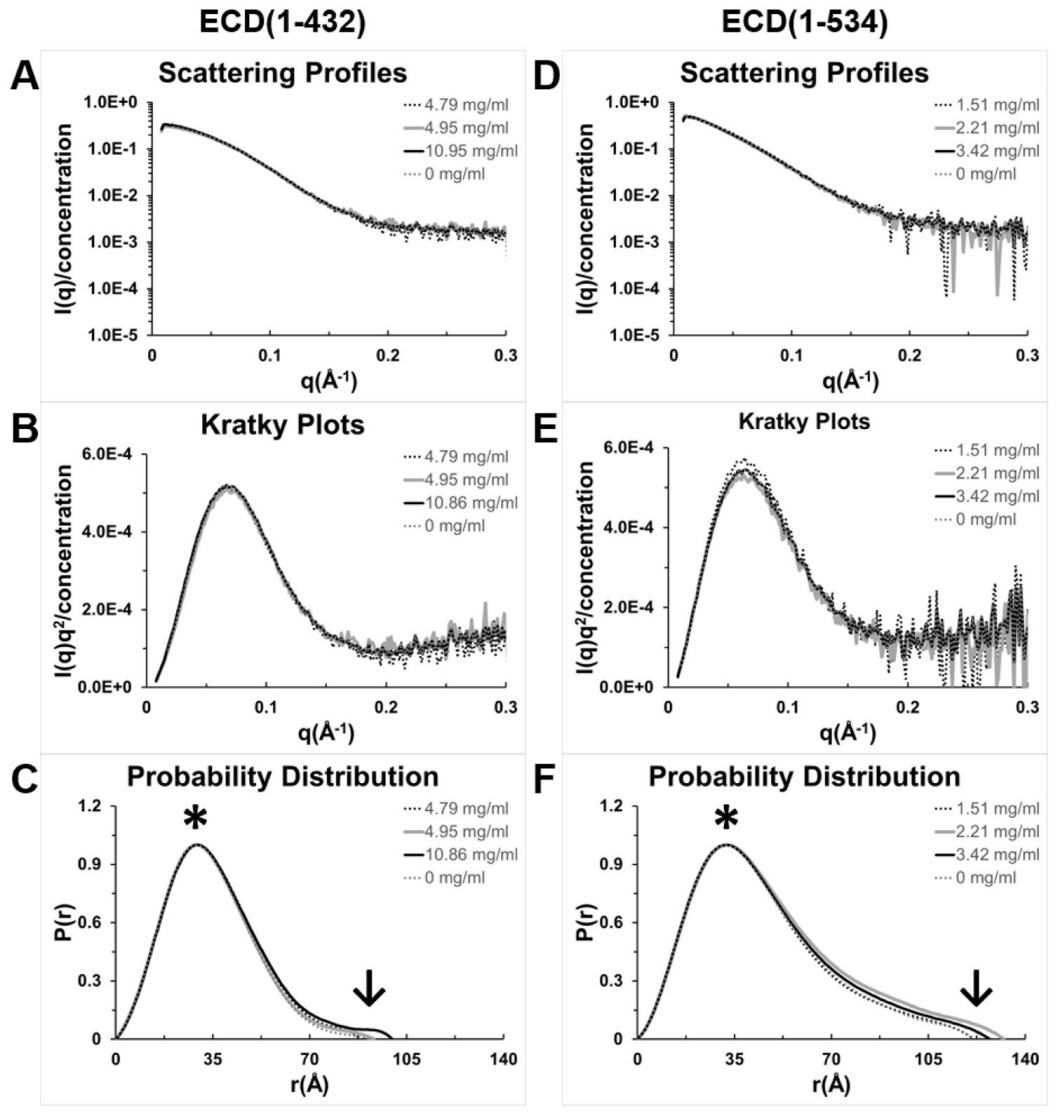
SAXS data and analysis for ECD(1–432) and ECD(1–534). Values for the most common interatomic distance are indicated with an asterisk and the maximum dimension (Dmax) with an arrow.

Thousands of crystallization experiments of the ECD fragments produced only clear drops or precipitation. Perhaps this was due to the flexibility of the fold. Therefore, we performed small angle X-ray scattering (SAXS) measurements to determine a low resolution ECD structure. SAXS is commonly used for the investigation of conformation, shape, and dimension of biopolymers in solution (Figure 3). Data were collected over three concentrations. The concentrations used were required to be from monodisperse samples as defined by DLS (dynamic light scattering) analysis [27]. Note that higher protein concentrations could be achieved for ECD(1–432) and thus the SAXS curves had better signal to noise. The scattering curves indicate strong data to q of about 0.2 for ECD(1–432) or 0.15 Å^−1^ for ECD(1–534) (Figure 3A & D), where q = 4πsin(θ)/λ and 2θ is the angle between the incident and scattered radiation of wavelength λ. Data beyond q of 0.3 (not shown) are weaker and noisy but still useful in the calculations according to Shanum software which gave optimal q of 0.65 for ECD(1–432) and 0.67 for ECD(1–534) [21]. All data were included in calculations.

**Figure 4:**
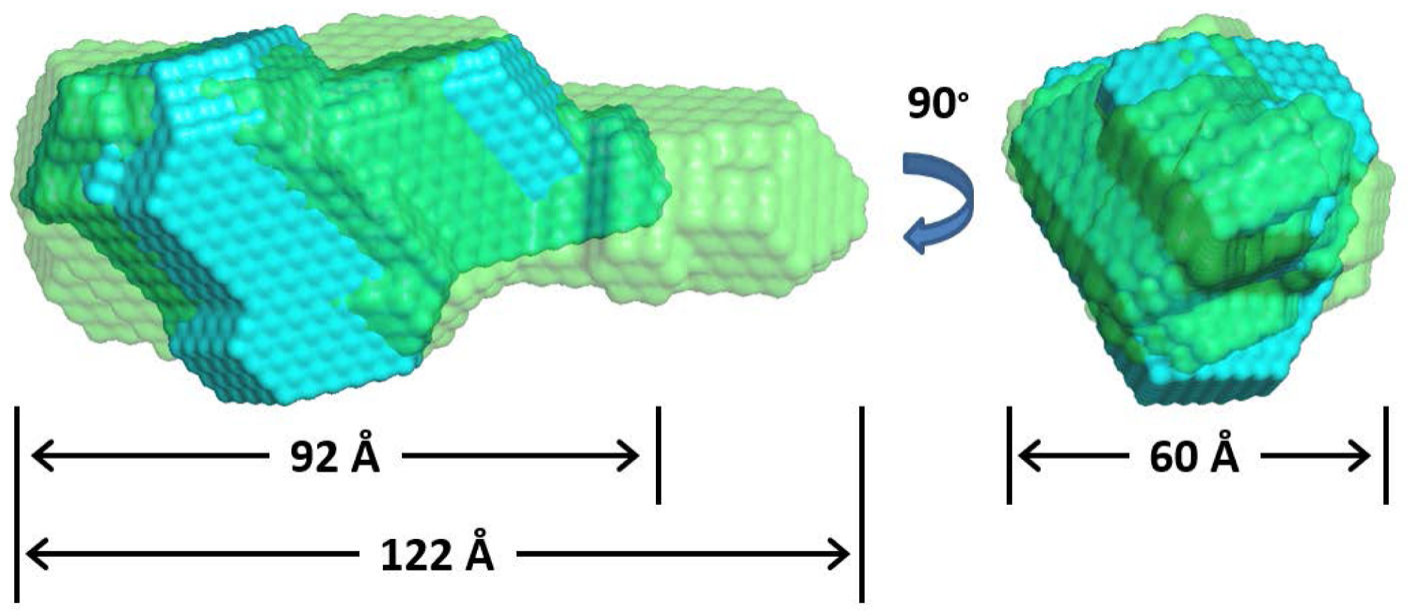
*Ab initio* bead models of ECD(1–432) (blue) and ECD(1–534) (green) with the lowest NSD values. For ECD(1–432) the best NSD value was 0.606 and from 9 models the maximum value was 0.675, mean was 0.633 and standard deviation was 0.023. For ECD(1–534) the best NSD value was 0.636 and from 9 models the maximum value was 0.750, mean was 0.665 and standard deviation was 0.041.

Analysis of the scattering curves using the Guinier approximation gives information about the radius of gyration (Rg). ECD(1–432) has an Rg of 26.3 Å and ECD(1–534) has Rg of 34.8 Å (Table 1). Presentation of the scattering data in the form of a Kratky plot provides information about the globularity (packing density) and conformation of a protein [29]. An unfolded protein will tend toward a horizontal asymptote while a well-folded globular protein will reach a peak and return to zero; a partially folded protein will be intermediate, reaching a peak, but not returning to zero and tending to increase at higher q values. Kratky plots of both fragments of ECD (Figure 4B & E) shows that they are both globular and folded.

The probability distribution function, P(r), plot gives the pairwise distance between atoms and the maximum dimensions (Dmax) for the molecule at the X-intercept (see arrow Figure 3C & F). The majority of ECD residues are within 30 to 35 Å of each other (asterisk Figure 3C & F). For ECD(1–432) the maximum dimension is 89–100 Å and for ECD(1–534) it is 121–132 Å (Table 1). *Ab initio* bead models for the molecular envelop were fit to the P(r) function [29] and the relative topology of the different domains was observed (Figure 4). The bead models show that the first 400 residues are globular and the next 100 residues are in an extended cylindrical structure. It is noteworthy that the C-terminus of ECD contains the CK2 consensus phosphorylation sites, Rb binding site, and PIH1D1 binding sites. These results show that ECD(1–432) forms the base of the scaffold that tethers various complexes to ECD. We calculated models using Gasbor software and obtained similar results (see Supplementary Figure 2A).

### 3.4. Molecular modeling of ECD(1–432) theoretical structures and triage using SAXS scattering data

Theoretical models for ECD(1–432) were calculated using molecular modeling tools and the best model was selected using the SAXS scattering data (Figure 3A). The I-TASSER webserver was used to predict structures. The I-TASSER method was introduced as a webserver after winning a community-wide protein structure prediction contest [30]. In addition to being a state-of-the-art prediction method, I-TASSER was chosen because it is completely automated, freely available online, has been used extensively, and its predictions come with reasonable estimates of model accuracy. Furthermore, it can be used to predict the structure of protein sequences as large as 1,500 amino acids by simply providing a single protein sequence in FASTA format and returns predicted models within 48 hours or less. For complete details of the methodology, please refer to the original publications [30–33]. I-TASSER uses a composite homology/*ab initio* methodology, wherein the structures of parts of the target sequence that can be confidently aligned to sequences of known structure are predicted using homology modeling, and *ab initio* loop modeling is used to fill in the gaps. I-TASSER also uses the structure-function paradigm to predict the biological function of a protein based on local and global similarity of the predicted structure to proteins of known structure and function.

I-TASSER makes use of PSIPRED [34,35] to predict the secondary structure of the target sequence. ECD(1–432) was predicted to be 47% helix, 11% beta-sheet, and 42% random coil, and is in good agreement with CD data (Figure 1B). To perform tertiary structure prediction, I-TASSER identifies template structures using multiple threading programs and takes only the most highly significant match from each method and ranks them according to each threading method’s past performance in benchmark experiments. The top template match for ECD(1–432), generated by the FFAS-3D threading algorithm [36], is PDB ID 4xgcA. Despite being the top match, ECD(1–432) and 4xgcA have a low sequence identity in the aligned region (0.13). Another measure of threading quality is the Z-score, where Z-scores greater than 1 indicate a good alignment. ECD(1–432) has a Z-score of 1.01 when threaded over 4xgcA. The highest threading Z-score among all of the top 10 templates (1.40) was obtained by threading the ECD(1–432) sequence over the second ranked template, PDB ID 5bq9A, which was identified by the PROSPECT2 threading algorithm [37]. The identities of the other 8 top templates range from 0.08 to 0.11, and the Z-scores range from 0.58 to 1.37. Taken together, these results indicate that ECD(1–432) is a hard target for protein structure prediction due to its large size, which complicates pure *ab initio* modeling, and lack of good structural templates, which complicates homology modeling. Finally, I-TASSER provides confidence scores (“C-scores”) for the top 5 predicted 3D models. C-scores can range from −5 to 2, with a C-score of greater than −1.5 being typically indicative of an overall correctly predicted global topology. In the case of ECD(1–432), the C-scores of the top 5 predicted models ranged from −3.42 to −2.39, all of which fall outside the range of C-scores that are usually taken to indicate a good overall prediction. The top 2 models deviated by 2.7 and 1.5 Å, respectively, from their template structures 4xgcA and 5bq9A [38].

**Figure 5:**
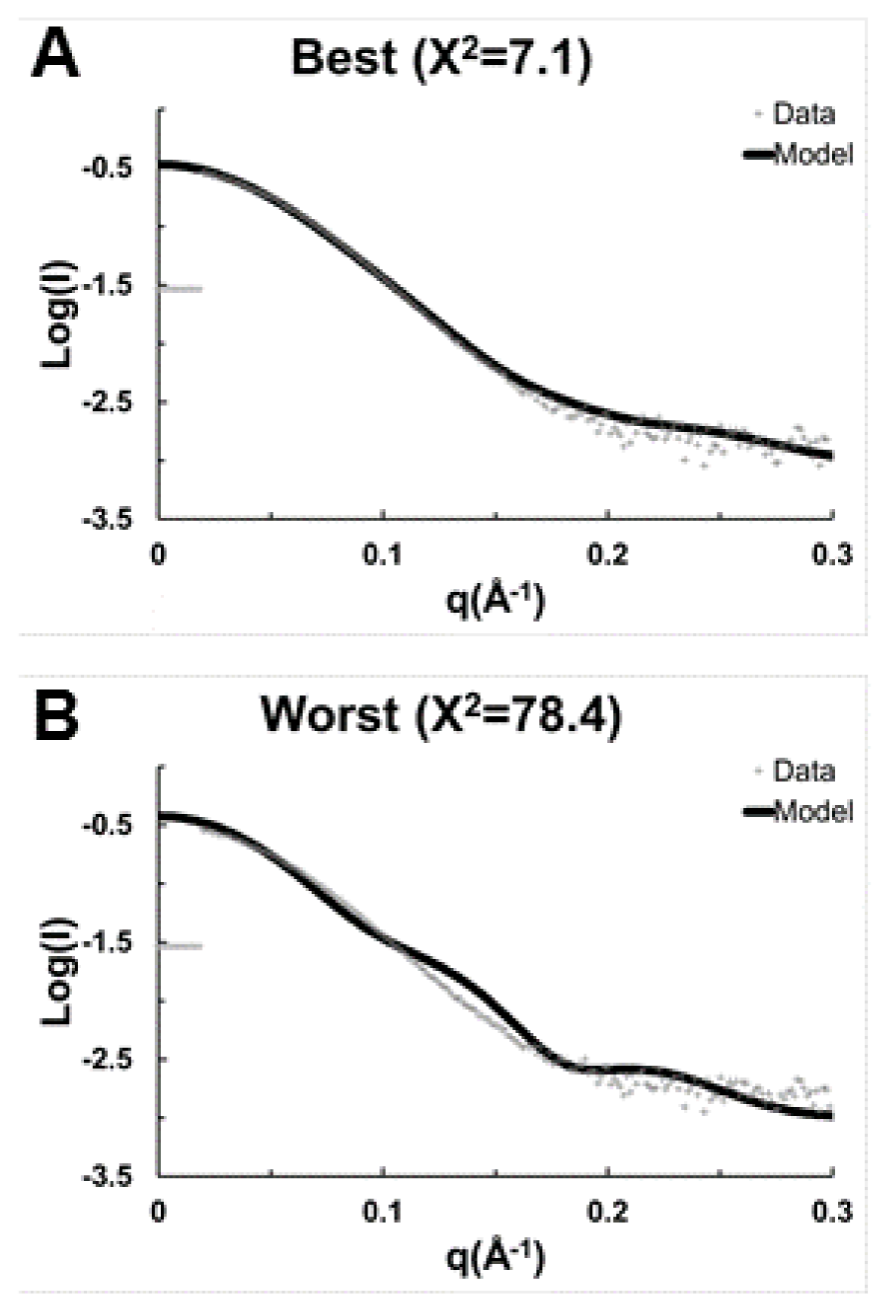
Representative samples of SAXS data calculated using the ECD(1–432) theoretical atomic models compared to the observed SAXS data; (A) the best model threaded with PDB ID 5bq9A, and (B) the worst model.

Because no single model stood out, all 5 top models were used to calculate theoretical SAXS scattering curves, and these curves were compared to the available experimental data (Figure 5). Atomic models were fit against the scattering data using Crysol [39]. Model 2, created by threading over 5bq9, had the best fit to the SAXS data (Figure 5A). Model 2 had a nice fit to the data from q = 0.0–0.2 Å^−1^ and reasonable fit from q = 0.2–0.3 Å^−1^ relative to the noise in the SAXS data. The calculated data for the worst model against the observed SAXS data is shown for comparison (Figure 5B).

**Figure 6:**
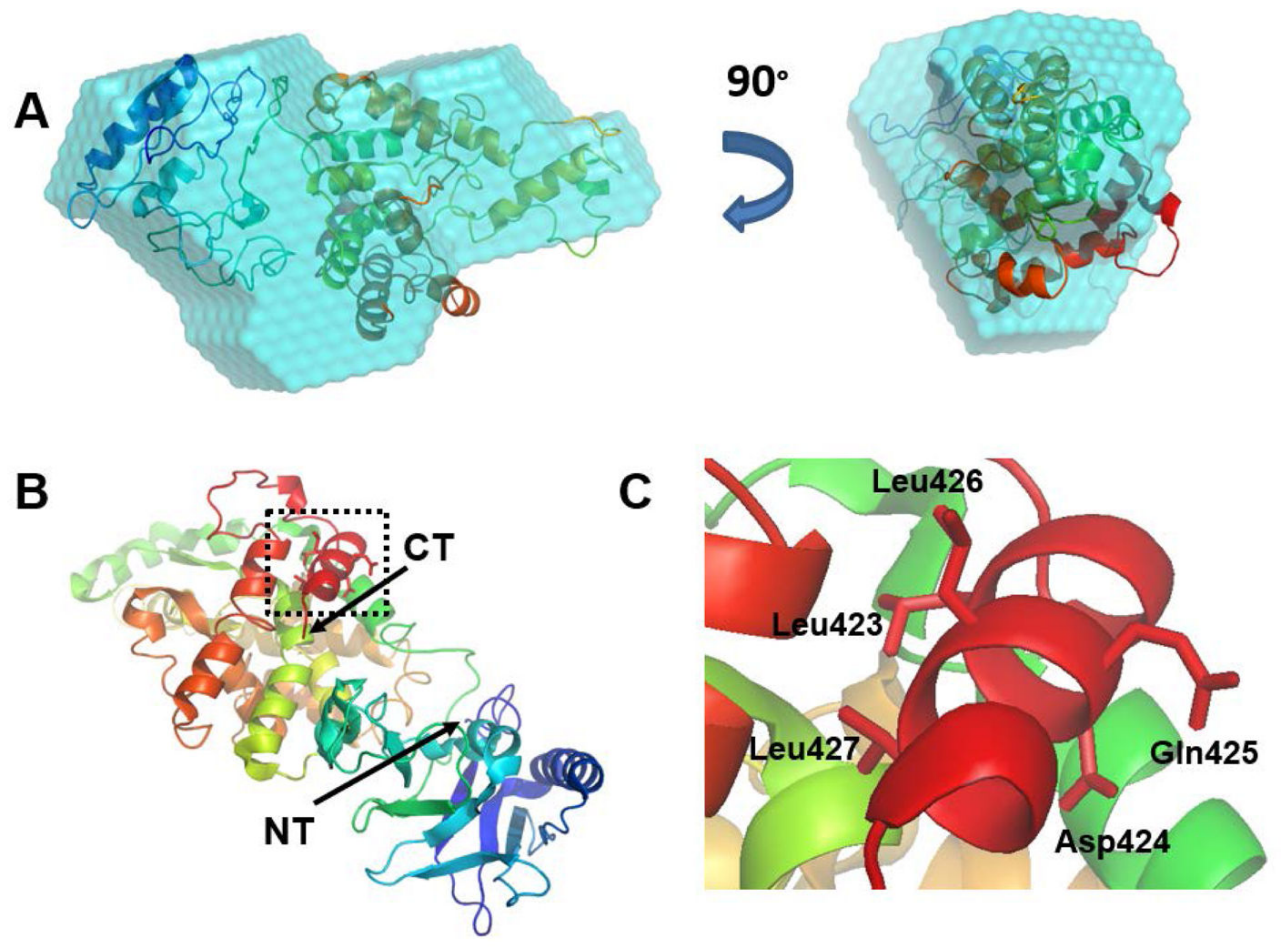
The best theoretical model of ECD(1–432). (A) Docked into the *ab initio* bead model using Situs software. (B) Ribbon drawing of the bilobal domain structure rainbow colored from N-terminus (Blue) to C-terminus (red); and (C) zoom-in on the C-terminal LxxLL motif (dotted box in part B).

The best theoretical model for ECD(1–432) was docked into the best ab initio bead model (lowest NSD) using Situs (Figure 6). The second model threaded with PDB ID 5bq9 gave the best fit to the bead model. Please note, 5bq9 is an uncharacterized protein lpg1496 from *Legionella pneumophila* subspecies *pneumophila*. So this threading experiment does not yield further clues into ECD function at this point in time. The secondary structure of this model (Supplementary Figure S1 line 3) is also in good agreement with the predicted secondary structures (Supplementary Figure S1 line 2) and predicted disordered regions (Supplementary Figure S1 line 4). The N-terminus of this model is positioned approximately where the N-terminus of ECD is most likely to be located from the interpretation of the SAXS bead models of ECD(1–432) and ECD(1–534) (Figure 4). The model fills the Dammin SAXS bead model well and has similar dimensions (Figure 6A). The model overlaid with the Gasbor SAXS model gives similar results (see Supplementary Figure 2B). The model has two domains composed of helices and strands with a flexible linker between them (Figure 6B). Of particular interest is the LxxLL motif. It is located on a helical region near the C-terminus (Figure 6C). This helical structure is probably loosely tethered and becomes elongated upon interaction with other proteins, as observed in the crystal structure of other proteins with the LxxLL protein-protein interaction peptide motif [40]. It may extend into the cylindrical region observed in the ECD(1–534) bead model (Figure 4).

## 4. Conclusions

CD, thermal, and SAXS analysis of ECD(1–432) and ECD(1–534) reveals that they have a folded globular structure that is loosely held together. The Kratky plot and the *ab initio* bead models show a globular structure for each protein and ECD(1–534) includes an extended cylindrical structure. The “best” theoretical model, produced by I-TASSER calculations, appears reasonable and, in the future, will be tested experimentally (e.g. FRET). This extended structure houses the Rb binding site and some of the CK2 sites where PIH1D1 binds (Supplementary Figure S1). Based on these results we conclude that ECD may form a base, or scaffold, for tethering of various protein partners and thus be a critical component for the formation of these complexes. This is consistent with the idea that in partially-condensed proteins the structured N-terminal domain (SNTD) organizes the remaining protein chain by intramolecular interaction [41]. Moreover an interspersed pattern of SNTD-docked regions and free loops can coordinate the assembly of sub-complexes in a defined loop section and enable novel regulatory mechanisms, one of which may be phosphorylation of docked regions.

## Supplementary Material



## Figures and Tables

**Table 1:** SAXS Data Collection and Processing Statistics for ECD(1–432) and ECD(1–534).

Sample	ECD(1–432)				ECD(1–534)			
Concentration (mg ml^−1^)	4.79	4.95	10.86	∞^−1^	1.51	2.21	3.42	∞^−1^
I(0) (Normalized concentration; cm^−1^)	0.33	0.32	0.35	0.32	0.52	0.54	0.52	0.50
Rg Guinier (Å)	27.7	27.2	28.5	26.3	34.7	37.8	36.3	34.8
Rg P(r) (Å)	27.3	27.0	28.5	26.4	35.6	38.8	37.1	35.6
Dmax (Å)	89.0	93.0	100.0	92.0	121	132	127	122
Porod volume (Å^3^)	90,302	86,625	93,368	87,266	114,882	126,440	117,895	116,206
DAM volume (Å^3^)	109,400	104,900	111,100	105,300	145,700	159,300	149,200	145,400
MW Porod (kDa)	53.1	51.0	54.9	51.3	67.6	74.4	69.4	68.4
MW DAM^a^ (kDa)	54.7	52.5	55.6	52.7	72.9	79.7	74.6	72.7

a where DAM is Dummy Atom Model

## References

[1] Hanahan D, Weinberg RA (2000). The hallmarks of cancer. Cell.

[2] Hanahan D, Weinberg RA (2011). Hallmarks of cancer: the next generation. Cell.

[3] Frolov MV, Dyson NJ (2004). Molecular mechanisms of E2F-dependent activation and pRB-mediated repression. J Cell Sci.

[4] Dyson N (1998). The regulation of E2F by pRB-family proteins. Genes Dev.

[5] Kim JH, Gurumurthy CB, Naramura M (2009). Role of mammalian Ecdysoneless in cell cycle regulation. J Biol Chem.

[6] Henrich VC, Livingston L, Gilbert LI (1993). Developmental requirements for the ecdysoneless (ecd) locus in Drosophila melanogaster. Dev Genet.

[7] Gaziova I, Bonnette PC, Henrich VC (2004). Cell-autonomous roles of the ecdysoneless gene in Drosophila development and oogenesis. Development.

[8] Zhang Y, Chen J, Gurumurthy CB (2006). The human orthologue of Drosophila ecdysoneless protein interacts with p53 and regulates its function. Cancer Res.

[9] Zhao X, Mirza S, Alshareeda A (2012). Overexpression of a novel cell cycle regulator ecdysoneless in breast cancer: a marker of poor prognosis in HER2/neu-overexpressing breast cancer patients. Breast Cancer Res Treat.

[10] Dey P, Rachagani S, Chakraborty S (2012). Overexpression of ecdysoneless in pancreatic cancer and its role in oncogenesis by regulating glycolysis. Clin Cancer Res.

[11] Bele A, Mirza S, Zhang Y (2015). The cell cycle regulator ecdysoneless cooperates with H-Ras to promote oncogenic transformation of human mammary epithelial cells. Cell Cycle.

[12] Horejsi Z, Stach L, Flower TG (2014). Phosphorylation-dependent PIH1D1 interactions define substrate specificity of the R2TP cochaperone complex. Cell Rep.

[13] Kakihara Y, Houry WA (2012). The R2TP complex: discovery and functions. Biochim Biophys Acta.

[14] Boulon S, Bertrand E, Pradet-Balade B (2012). HSP90 and the R2TP co-chaperone complex: building multi-protein machineries essential for cell growth and gene expression. RNA Biol.

[15] Horejsi Z, Takai H, Adelman CA (2010). CK2 phospho-dependent binding of R2TP complex to TEL2 is essential for mTOR and SMG1 stability. Mol Cell.

[16] Mir RA, Bele A, Mirza S (2015). A novel interaction of ECD protein with R2TP complex component RUVBL1 is required for the functional role of ECD in cell cycle progression. Mol Cell Biol.

[17] Suh HW, Yun S, Song H (2013). TXNIP interacts with hEcd to increase p53 stability and activity. Biochem Biophys Res Commun.

[18] Claudius AK, Romani P, Lamkemeyer T (2014). Unexpected role of the steroid-deficiency protein ecdysoneless in pre-mRNA splicing. PLoS Genet.

[19] Andrade MA, Chacon P, Merelo JJ (1993). Evaluation of secondary structure of proteins from UV circular dichroism spectra using an unsupervised learning neural network. Protein Eng.

[20] Petoukhov MV, Franke D, Shkumatov AV (2012). New developments in the program package for small-angle scattering data analysis. J Appl Crystallogr.

[21] Konarev PV, Svergun DI (2015). A posteriori determination of the useful data range for small-angle scattering experiments on dilute monodisperse systems. IUCrJ.

[22] Chacon P, Wriggers W (2002). Multi-resolution contour-based fitting of macromolecular structures. J Mol Biol.

[23] Wriggers W (2010). Using Situs for the integration of multi-resolution structures. Biophys Rev.

[24] Li X, Romero P, Rani M (1999). Predicting Protein Disorder for N-, C-, and Internal Regions. Genome Inform Ser Workshop Genome Inform.

[25] Romero P, Obradovic Z, Dunker K (1997). Sequence Data Analysis for Long Disordered Regions Prediction in the Calcineurin Family. Genome Inform Ser Workshop Genome Inform.

[26] Romero P, Obradovic Z, Li X (2001). Sequence complexity of disordered protein. Proteins.

[27] Borgstahl GE (2007). How to use dynamic light scattering to improve the likelihood of growing macromolecular crystals. Methods Mol Biol.

[28] Garnier J, Gibrat JF, Robson B (1996). GOR method for predicting protein secondary structure from amino acid sequence. Methods Enzymol.

[29] Putnam CD, Hammel M, Hura GL (2007). X-ray solution scattering (SAXS) combined with crystallography and computation: defining accurate macromolecular structures, conformations and assemblies in solution. Q Rev Biophys.

[30] Zhang Y (2008). I-TASSER server for protein 3D structure prediction. BMC Bioinformatics.

[31] Roy A, Kucukural A, Zhang Y (2010). I-TASSER: a unified platform for automated protein structure and function prediction. Nat Protoc.

[32] Yang J, Yan R, Roy A (2015). The I-TASSER Suite: protein structure and function prediction. Nat Methods.

[33] Yang J, Zhang Y (2015). I-TASSER server: new development for protein structure and function predictions. Nucleic Acids Res.

[34] Buchan DW, Minneci F, Nugent TC (2013). Scalable web services for the PSIPRED Protein Analysis Workbench. Nucleic Acids Res.

[35] Jones DT (1999). Protein secondary structure prediction based on position-specific scoring matrices. J Mol Biol.

[36] Xu D, Jaroszewski L, Li Z (2014). FFAS-3D: improving fold recognition by including optimized structural features and template re-ranking. Bioinformatics.

[37] Xu Y, Xu D (2000). Protein threading using PROSPECT: design and evaluation. Proteins.

[38] Roy A, Yang J, Zhang Y (2012). COFACTOR: an accurate comparative algorithm for structure-based protein function annotation. Nucleic Acids Res.

[39] Svergun DI, Barberato C, Koch MHJ (1995). CRYSOL - a Program to Evaluate X-ray Solution Scattering of Biological Macromolecules from Atomic Coordinates. J Appl Cryst.

[40] Zanier K, Charbonnier S, Sidi AO (2013). Structural basis for hijacking of cellular LxxLL motifs by papillomavirus E6 oncoproteins. Science.

[41] Simister PC, Schaper F, O’Reilly N (2011). Self-organization and regulation of intrinsically disordered proteins with folded N-termini. PLoS Biol.

