# Supplementary material for: Biophysical characterization and modeling of human Ecdysoneless (ECD) protein supports a scaffolding function
Source: AIMS Biophys. Author manuscript; Available in PMC 2017 May 8. (PMC5421643; doi:10.3934/biophy.2016.1.195)
Supplement: Supplementary file 1 [file NIHMS821895-supplement-supplement_1.pdf]

AIMS Biophysics

AIMS Biophysics, 3(1): 195-208.
DOI: 10.3934/biophy.2016.1.195
Received: 09 December 2015
Accepted: 06 March 2016
Published: 09 March 2016

http://www.aimspress.com/journal/biophysics

Research article

Biophysical characterization and modeling of human Ecdysoneless (ECD) protein supports a scaffolding function

Riyaz A. Mir [1], Jeff Lovelace [2], Nicholas P. Schafer [3,] Peter D. Simone [2], Admir Kellezi [2], Carol Kolar [2], Gaelle Spagnol [4], Paul L. Sorgen [4], Hamid Band [1,2,4], Vimla Band [1,]*, and Gloria E. O. Borgstahl [2,4,]*

[1] Department of Genetics, Cell Biology and Anatomy, University of Nebraska Medical Center, Omaha, NE 68198, USA
[2] Eppley Institute for Research in Cancer and Allied Diseases, University of Nebraska Medical Center, Omaha, NE 68198, USA
[3] Interdisciplinary Nanoscience Center (iNANO), Aarhus University, Gustav Wieds Vej 14, 8000 Aarhus C, Denmark
[4] Department of Biochemistry & Molecular Biology, University of Nebraska Medical Center, Omaha, NE 68198, USA

* **Correspondence:** Email: vband@unmc.edu; gborgstahl@unmc.edu.

Supplementary materials

```
1   MEetmKlAtm EDtVEYcLFL IPDesrdsdk HkEILQkyIE RIiTrFAPmL 50
     aaaaaaaaaa aaaaaaaaaa caaaaaaaaa aaaaaaaaaa abaatcctcc
    CCCCCCCCCC CCSSSSSSSS CCCCCCCHHH HHHHHHHHHH HHHHHHHHHH
    DDDDDDDDDD D                     DDDDDDDD

51  VPYIWQNQPF NLKYKPgKGg VPAHmfGvTK FGDNIEDEWF IVYviKQITK 100
    bttttccctt ttttctttcc bbaaaaaaac cccccaaaaa aaaaaaaccc
    CCCCCCCCCC SSSSSSCCCC CCCSSSSSSS CCCCHHHHHH HHHHHHHHHH


101 EFPELVARiE DNDGEFLLIE AADFLPKWLD PeNStNRVFF chGELCIIPa 150
    caaaaaaaaa ttttaaaaaa aaaaaatccc cttttttaba ttttttbbac
    HCCCSSSSSS CCCCCHHHHH HHHHCCCCCC CCCCCCSSSS SCCSSSSSCC
        DDDDDD DDDD

151 PrKSgaeSWL PtTPPTIpQA LnIItaHsEk iLASESirAA VnRRirGYPE 200
    ctccccccttc ttcctccbaa aaaaaaaaaa aaaaaaaaaa aattttccct
    CCCCCCCCCC CCCCCCHHHH HHHHHCCCHH HHCCHHHHHH HHHHHHHHHH
```

```
        DDDDDD DDDDDDDDDD DDDD        DDDDDDDDD DDDDDDDDDD

201 kiqASLHRAH CFLPAGIvAV LKQrPRLvaA aVQAFYLRDP IDLRACRvFK 250
    aaaaaaaatt tacaaaaaaa aaacaaaaaa aaaaaattct taaaaaaatt
    HHHHCCCSSS SSCCHHHHHH HHHCHHHHHH HHHHHHCCCH HHHHHHHHCC


251 TFLPETRIMt SVTFTKCLYA QLVQQrFVPD RRSGYrLPPP sdPQYRAhEL 300
    cccaaaaaaa aaataaaaaa aaabbbbbct ttttttccct tcctaaaaaa
    CCCCCCCCSS SSSSCHHHHH HHHCCCCCCC CCCCCCCCCC CCHHHHHHHH
                                            DDD DDDDDDDDD

301 GMKLAHGFEI LCSKCSPHFS DckkSLVTaS PLWAsFLESL KkNDYFKGLi 350
    aaaaaaaaaa attttcttaa tttaaabbbc caaaaaaaaa aaaaaaaaca
    CCHHHHHHHH HHHHHHCCCC CCCCCCCCCC HHHHHHHHHH HHCCCCCCCC


351 eGSaQYrERL EMAeNYFQLS vdwPESSlAM SPGEEILTlL QTiPFDiedL 400
    aaaaaaaaaa aaaaaaaacc ccccaacccc caaaaaaaaa accaaaaaaa
    CCHHHHHHHH HHHHHHHHHH CCCCCCCCCC CCHHHHHHHH HCCCCCHHHH
          DDD             DDDDDDDDDD DDDDDDDDDD DDD    DDD

401 KkEaAnLPPE DDdqWLDLSP DQLDQLLQeA vGkKEsesvs keekeqNYDl 450
    aaaaaccccct aaaaaatccc taaaaaaaaa aaaaaaaaaa aaaaaaaaaa
    HHHCCCCCCC CCCHHHCCCH HHHHHHHHHH CC
    DDDDDDDDDD DDDDDDDDD       DDDDD DD

451 teVSeSMKAF ISKVStHKGA ElPRePSEAP ITFDADSFLN YFDKILGprp 500
    aaaaaaaaaa aaaaaccccc cccccataaa aaaaaaaaaa attttccccc

                      DDD DDDDDDDDDD D                   D

501 nESDSDDldd EDfEclDSDD DldfEthEpg eeaSlKGtLd nLKSYMAqMD 550
    tttttaaaaa aaaaaaccaa aaaaaaaaaa aaaaaaaaaa aaaaaaaaaa

    DDDDDDDDDD DDDDDDDDDD DDDDDDDDDD DDDDD

551 qELAHTcisk SFTTrnqveP vSqTtdnNsd EEDSGtGesv mApVDVDLNL 600
    aaaaaaaatt ttcccttccc bbtccccccc caaccaaaaa bbaaaaaaaa

                       DD DDDDDDDDDD DDDDDDDDDD DDDDDDDDDD

601 vSNILESYSS QAGLAGPASN LLqSMGVqLP DNtDHrPtsk ptkn       644
   aaaaattttc ttcccccaa aaatbbbccc ttttccttcc ctct

   DDDDDDDDDD DDDDDDDDDD DDDDDDDDDD DDDDDDDDDD DDDD
```

Figure S1. Ecd sequence analysis summary. The first row indicates the amino acid sequence of Ecd: capitols indicate residues that are identical between human, mouse and cow; lower case are homologous; underlined are not conserved. Second row the Garnier Robson predictions for alpha helix (a), beta (b), coil (c) and turns (t) are indicated. Third row alpha helix (H), beta (S), and coil (C) from molecular modeling of ECD(1–432) are indicated. Last row PONDR VL-XT disordered regions are indicated with D. The LxxLL residues 423–427 are highlighted in light blue; acidic residues 499–527 are highlighted in green; CK2 sites 503, 505, and 518 are highlighted in magenta, CK2 sites 572, 579, and 584 are highlighted in red. The ends of deletion constructs are highlighted in yellow. Note, there is a small deletion in mouse at 531–533 and a small insertion in mouse at 568–569.

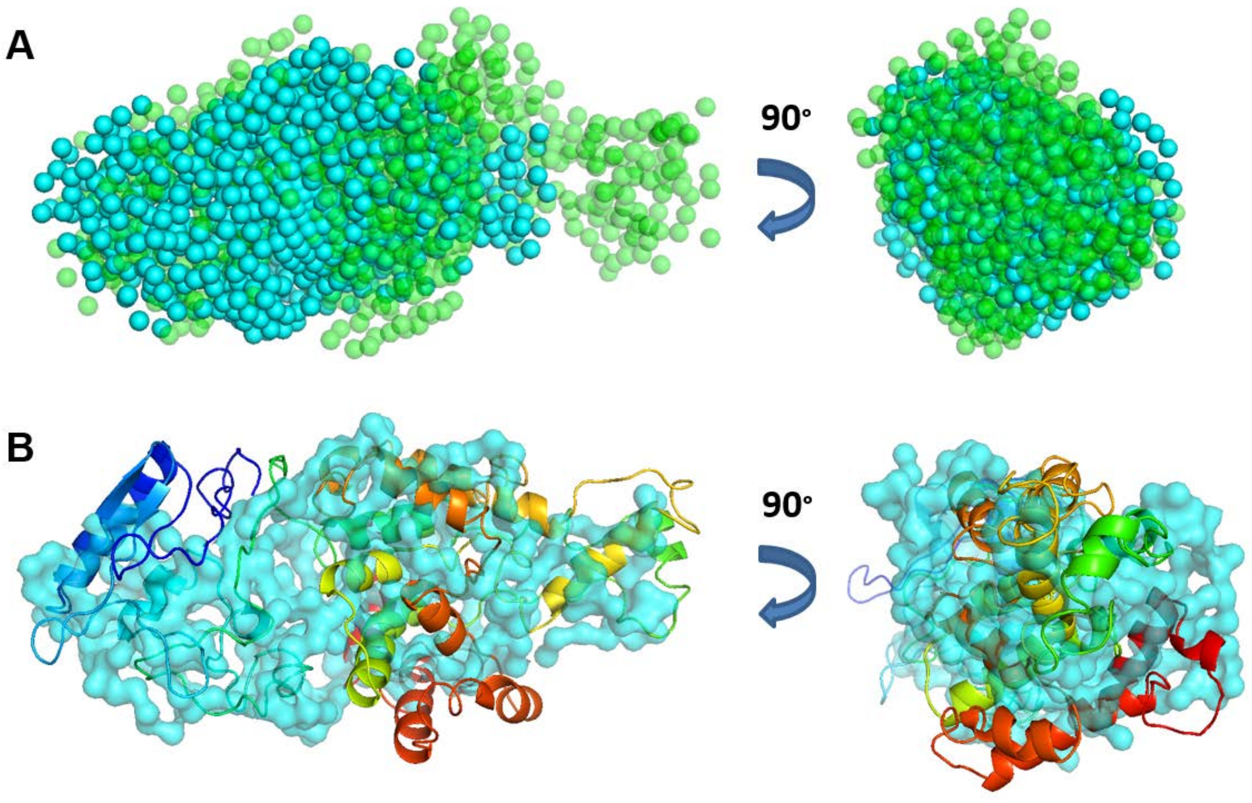

Figure S2. Gasbor *ab initio* calculations. (A) SAXS model for ECD(1–432) in blue and ECD(1–534) in green. For ECD(1–432) the best NSD value was 0.866 and from 6 models the maximum value was 1.060, mean was 0.932 and standard deviation was 0.098. For ECD(1–534) the best NSD value was 1.271 and from 6 models the maximum value was 1.414, mean was 1.271 and standard deviation was 0.137. (B) The best theoretical model of ECD(1–432) docked into the GASBOR model using Situs software.

AIMS Press

© 2016 Gloria Borgstahl, et al., licensee AIMS Press. This is an open access article distributed under the terms of the Creative Commons Attribution License (http://creativecommons.org/licenses/by/4.0)
